# Diagnosis and Treatment of Benign Tumors

**Published:** 1882-10

**Authors:** Geo. Halsted Boyland

**Affiliations:** Baltimore, Maryland.


THE
AMERICAN JOURNAL
OF
DENTAL SCIENCE.

Vol. XVI. THIRD SERIES—OCTOBER, 1882. No. 6

### ARTICLE I.

### *Diagnosis and Treatment of Benign Tumors.*

BY GEO. HALSTED BOYLAND, A. M., M. D., ETC., OF BALTIMORE, MARYLAND.

A tumor, as the word signifies, is any swelling. But from this broad definition practical wants have led to the very many divisions, subdivisions and classifications that crowd our exhaustive works on surgery; indeed, this one subject not only claims more space than any other in them, but whole works are published continually on tumors as a class, or even on any one kind of tumor. Certainly this is perplexing. It is our aim, therefore, in the present article, to place before the general practitioner such condensed information on the subject of benign tumors as may be useful in ordinary practice.

Under the term new growth, foreign growth, in its widest sense, are comprehended all those foreign processes that take place in the human body; either by cell formation, or by precipitates and concretions out of the liquids. The latter possess no texture; these are mostly stones and irregular concretions. On the other hand, we may readily recognize a regular structure and texture in the first named, which, in chemical relation also, claim for them the designation of "organic;" moreover, they offer a long list of common characteristics, which in many respects are entirely independent of the tissue or part in which they appear.

These organic new growths fall, of themselves, into two groups, benign and malignant, principally the former claiming attention in this paper. In spite of the great variety of organic new growths, they all resemble each other in one respect, and that is, that they are composed of the same morphological elements that we find also in the healthy body, and develop themselves on the same plan of construction as normal tissue. In the arrangement and length of development of their elementary principles, the different new growths vary perceptibly as well from normal organs, as from each other. It is upon this and upon the different texture of the pseudoplasma that the distinction above named founds itself, of which more anon.

It is of great importance, theoretically as well as practically, that the morphological elements of new growths can be subject to the same changes as those of the normal organs of the body—namely, the so-called regressive metamorphosis: the fatty, colloid, amyloid degenerations, the mucous softening, the chalky (lime) and bony processes. The chemical composition of new growths shows no marked differences from that of normal tissue.

Albumen, fibrin, cheesy matter, fat, further, substances that give lime and cartilaginous lime on boiling, pigment, also the ordinary salts, cooking salt and phosphate of lime, and finally, water, in greater or less quantity, have been with certainty demonstrated, by chemical analysis, to exist in tumors. In tumors we often find widening and tearing of the blood vessels that run through them, therefore, also, bleeding, inflammation, ulceration and gangrenous disturbance are also found.

*Ætiology.*—The general ætiology of new growths is very dark. As local cause, only a certain irritation of the diseased part can be considered. As regards predisposition, it may be noticed that new growths are not unfrequently inherited; sometimes they are on the patient when born; again, they are at other times endemic or epidemic. Predisposing are also early youth and old age, overwork and insufficient food, also other disturbances of the general system, brought about by former diseases. But to return to our two groups—embracing all tumors known—benign and malignant.

From a practical standpoint those tumors are benign which rest upon purely local causes and interfere with certain functions only by pressure upon or pushing aside organs. They can entirely stop the action of an organ, and thus become harmful to the individual, even, perhaps, threatening life, but do not at all exercise any evil influence directly upon the general system. Finally, they remain circumscribed in the locality where they sprung up, and do not return after extirpation. In distinction from these we designate as malignant tumors, those that continually and steadily grow, and finally soften (break down); that successively appear in different places and different organs; that return upon the same or other places after extirpation (recidiration), finally, that develop a marked and general affection (cachexia), and most always bring death to the individual suffering from them.

To the first belong fatty tumors (lipoma), sebaceous tumors (steatoma), fibrous tumors (fibroma), the different skin tumors (cysts, lupiæ) and bone growths (exostoses); vascular tumors (aneurisms, varices, telangiectasis), also polypi, cartilaginous tumors (enchondroma), that first became distinguishable by microscopic research, and were earlier mostly classed under the so-called steatoma, but only on account of their consistency and outward appearance. The representative of malignant tumors, from Hippocrates down to the present day, is cancer (carcinoma), so that malignant and cancerous are still used as synonymous. The fungus medullaris can be only technically a distinct or separate form of cancer; its origin being a hardened mass (scirrhus). Sarcoma is also included, and the tubercle, that formerly belonged to inner medicine only in the category of malignant neoplasms.

*Diagnosis and Treatment of Fibroma.*—The diagnosis of fibroma is not without difficulty. Fibrous tumors can be confounded with other hard tumors, or even some soft ones; in many cases it is, for instance, difficult to say wit] certainty whether the tumor is more of a fibrous or sarcomatous nature. The diagnosis, by a due regard to the tough, stringy or knotty consistency of the tumor and its hardness, must be our main *point d' appui* in excluding other growths, such as enchandroma,, osteoma, cavernous tumors, cysts, cancers and sarcoma. Fibrous tumors, like other benign ones, do not possess the tendency of malignant tumors, to soften from the centre out or to soften in different places. As opposed to malignant tumors, fibroids never cause disturbances of the general system, and generally never other disturbances than those of purely mechanical origin and corresponding to the size and locality of the tumor. Swelling of the adjacent lympth glands is absent, except when it is occasioned by inflammation of the tumor. Emaciation and loss of strength are also absent, as a rule, but can develop when the tumor by its position, by ulceration or hemorrhage of its ulcerated surface, produces disturbance of the general system. The only treatment of fibroid tumors consists in operative removal. In general, thorough and complete extirpation with the knife is the best; nevertheless, if the seat and relations of the tumor admit it, other means of separation may be advantageous—écrasement linèaric. Chloroform may be given. Prof. Nathan R. Smith used a little chloroform in the extirpation of a fibroid tumor of the breast, in a young colored girl. I had the pleasure and honor to assist him, at his request, in this operation. The patient, a young woman, was about 18 years of age and well nourished, otherwise perfectly healthy, menstruating regularly. The tumor was situated in the left breast, movable and hard. The Professor grasped the tumor at its base and made the incision along the upper surface with a scalpel, and in the long diamater, while I held the edge of the wound and took up the arteries. The operation lasted but a very few minutes; no sutures were used, the edges of the wound being secured by adhesive plaster, and the patient making a good and rapid recovery. The tumor was about the size of a turkey's egg. There was nothing peculiar about the case, but it is a good, typical one, illustrating the ordinary facility with which fibroids may be removed, and a guarantee thus given against return; and it further gives some idea of Prof. Smith's views upon the administration of chloroform during surgical operations. When the girl asked to be thoroughly narcotized, he replied, "I do not want to put you into your last sleep." We gave her, accordingly, a partial narcosis only. In the history of a given case of fibroid we will generally find the growth of the tumor has been slow and without pain.

*Diagnosis and Treatment of Lipoma.*—Fatty tumors develop themselves almost entirely at an advanced age. The acceptance of the diathesis lipomatosa theory is not warranted by practical observation. The etiology is dark. A blow, push or knock may cause them. They grow slowly and without pain to the patient, whose first inconvenience from them is felt when they attain a large size. To the hand they fell like lumps of normal adipose tissue, that can be pushed aside, and have always a certain tough resistance. The diagnosis of this particular kind of tumor is comparatively easy. Pharmaceutical treatment is almost useless. Extirpation is the only radical cure, and this should be done as early as possible. If the tumor has attained the size of a nut or of a hen's egg, or is growing in such a manner as may be seen from day to day, then the operation should be performed at once, as it is very simple, and certain success always follows it at this period, while longer delay invariably brings some dangers. But if the base of the tumor has already passed the size of a hand, then it is better, as a rule, not to operate; because such a lipoma, left to itself has never had death as a consequence, while an operation that would leave a wound surface of such extent, even undertaken in the most favorable conditions, may endanger life. If the tumor threatens to break through the skin, the operation should be performed, under almost all circumstances; also when the seat of the tumor endangers life. The extirpation of fatty tumors requires great care. At times it is impossible to remove all parts of a lipoma. But even if we remove the most of it we have accomplished much, and it is pretty well established that pieces left behind neither become the germs of larger tumors nor interfere in any way with cicatrization.* In the case cited by Burow,† who extirpated an enormous growth of this class, weighing 27½ pounds, it was impossible to remove the whole tumor. "The healing of the wound took place with incredible rapidity, although large pieces of the still remaining lipoma died in the wound and sloughed off." Considerable hemorrhage may occur, but this is exceptional. If the patient is knife-shy, the écraisement linéarie is in order, but the galvano-caustic wire is much better, and prevents effectually hemorrhage from smaller vessels. Sometimes it is possible, after making an incision in the skin, to isolate and tear out a lipoma with the fingers, by pulling the tumor toward the operator with one hand and pushing back the skin from around it with the other.

*Diagnosis and Treatment of Enchondroma.*—The cartilaginous tumor forms a round mass with smooth or slightly nodulated surface, that can attain different sizes; sometimes very large size. The commencement may be only as large as a pea, and can take place wherever cartilage exists in the body (Virchow) Not infrequently these tumori, later on, become ossified. They generally cause little trouble beyond pressure, and can exist from fifteen to twenty years as harmless tumors. The chemical examination of enchondroma has determined that they give chondrin as their main principle. As regards their

---

*Lebert. *Abhandlung der practischen chirurgie*, p. 119.

†Burrow. *Deutsche Klinik*, No 24.

etiology they often date from earliest childhood, and I. Müller notes especially that the mechanical influences of life and the process of formation of bone during childhood are the first causes of abnormal cartilaginous growths. As far as my own personal experience is concerned. I have generally seen them on the fingers and toes. One case of great interest merits a line in this connection. It was that of a young man of about 25 years of age, who had enchondroma of the size of black walnuts on all the fingers and toes of both hands and both feet. Operation was, of course, not thought of, and the patient, who lived in a small German town (Juebingen), came periodically to Baun's clinic, where I was practicing at the time, to be exhibited to physicians and students, more as a surgical curiosity than anything else.* Enchondroma feels hard to the hand, and their diagnosis on the living is not always easy. Even when they are situated on the periphery, and are thoroughly cartilaginous, they have a resistance that resembles that of bone: they feel as hard as stone, ivory-like, as a scirrhus of the breast or a true bony tumor (exostosis). This is exceptional, however. They generally possess a light, glandular, warty surface, of large size, and the nodules on the periphery appear to roll themselves inward, and not to go over directly on to the base of the tumor (soft parts or bones). Exostoses, on the contrary, have a smooth or jagged surface, and spread evenly on all sides in the bone substance. The differential diagnosis is more difficult as regards fibroids, as enchondroma can develop themselves, not only in solt parts, but also on bones and sometimes inside bones, and for this reason can be, covered with a bony capsule outwardly, formed by pushing up the bone from within.

The only successful treatment is operative. Even this is not always advisable, unless the tumor, by its position and

* In this case the tumors were situated on the last rows of phalanges, at the joints.

size, interferes with the function of important parts or threatens danger from expectant breaking out. According to its position, the extirpation may be made by the knife, or with saw and chisel, or we may amputate the part upon which the growth sits. The experience of Dieffenbach in this respect is very valuable, that even after partial extirpation cicatrization can follow. The ossification of pieces of enchondroma left in the wound is regarded as fixed.* If part of the tumor is soft, if it is combined, or is a cystochondroma, we can never count upon anything but a total extirpation. If large arteries run through an enchondroma we may have great difficulty in arresting hemorrhage, in making a partial extirpation, as it is impossible to get hold of the vessel with the pincette, to tie it. As it lies imbedded in the cartilaginous substances, our only recourse is cautery with the iron (actuale); even this does not always suffice. The galvano-caustic would be more likely to meet our wants here than almost any other method.

*The Diagnosis and Treatment of Exostosis.*—Naturally follows, just as enchondroma may become exostoses, and these latter pathologically have the same distinguishing features as enchondroma. We have a soft, porous, spongy exostosis, a hard, ivory-like exostosis, and osteo-cartilaginous exostosis. Exostosis can exist on any bone. It is usually of slow growth, and the soft parts covering such a bone remain healthy. There is no pain during the earlier stages of growth, or even later, unless the exostosis, having attained a very great size, presses severely upon the soft parts, when inflammation may follow, bringing ulceration of the skin, and this in turn succeeded by laying bare the exostosis. Rokitansky and Nélaton attribute the ending in necrosis only to the hard, smoothy-ivory-like growth (exostosis eburnea), probably on account of the great dearth of canals for the blood vessels—Haversian canals. Any of the forms of exostosis may become inflammatory, suppurative and carious. They probably rest upon a dyscrasia, or

* Lebert Loc. cit.

are often hereditary. They may be only one, or many, and on different parts of the body. There is but little more to be said as regards these immovable, hard, jagged, or smooth, bony tumors—in so far as palpation is concerned; there is less that will aid us in making a diagnosis of exostoses if they cannot be reached by the hand, on account of lying deep and being covered with soft parts; for if undoubted exostosis do not exist on other parts of the body, and thus lead us on the right track, we may as justly attribute the functional trouble to a tumor of another class. In comparison with enchondroma, the surface of an exostosis is smoother, less nodulated, but often sharp like a cock's comb. The treatment of exostosis is always difficult, sometimes dangerous, often useless. We may only expect results from antiphlogistic and anti-dyscrasic means, when we have to do, not with a true exostosis, but with an ostephyte still in process of formation; that is, with an inflammation of the bone or periosteum. True exostosis can only be removed by surgical operation. If its position is such that the operation would be more dangerous than the further growth of the tumor, it should be left untouched. If the patient desires an operation on account of deformity, or great pain, or interference with the use of parts, it may be performed, provided there is no special danger. Nevertheless, no matter what plan is adopted for the removal of an exostosis, there are all the dangers of a deep-seated suppurration, with affection of the bone to follow; osteo-myelitis, ending in pyæmia, after simple removal of an exostosis, has been frequently observed.

The operation for exostosis, consists: 1st, in sawing off, *i. e.*, extirpation of the tumor; 2d, the employment of actual cautery; 3d. resection of the part of the bone on which the exostosis sits; 4th, amputation of the part affected. The ordinary operation, that is, the one that the general practitioner will oftenest have to perform, consists in laying bare, fully and freely, the exostosis by a long incision, the skin being held back by hooks, one on either die, in the hands of an assistant; the tumor is then sawed off with the saw, chain saw, or removed by chisel and hammer, care being taken to hold the chisel well down, parallel to the bone. In vascular, spongy exostosis, with broad base, the best method is the pereum caudens, as it gives us a guarantee against profuse bleeding. Only under these circumstances should actual cautery be employed in the removal of exostosis, as it always causes a partial necrosis, followed by inflammation and suppuration. In order to prevent this, it has been proposed to lay bare the exostosis, carefully scrape off the periosteum, and leave the rest to nature. Necrosis of the upper layer may be, of course, expected after this, but it is extremely doubtful if the whole tumor could be sloughed off in this way, Osteoma of the soft parts must be extirpated like other tumors.

*Diagnosis and Treatment of Telangiesasis, Fungus Vasculosis, Mœvus.*—These are mostly congenial, sometimes caused by a blow, push, or pressure. Mostly on the outer skin; they may, however, appear on mucous membranes; for example, on the lips. Diagnosis generally easy. They may exist on all parts of the body, varying in color from a bright red to a dusky purple and dark brown, in size from a pin's head to that of a black walnut. They have been mistaken for epithelioma, aneurism, lipoma and varices, but these are extreme exceptions, not likely to be met with in general practice; the characteristics of the tumors mentioned being usually so very marked as to make a mistake difficult; besides, they belong to a more advanced period of life. The forehead is often chosen as their seat. Simple angioma is never malignant, even though local recidiration may occur after incomplete extirpation. My plan is to let a nævus alone, unless it grows rapidly. From the long list of therapeutical and operative measures, the following will be found most useful: Compression by bandage, application of cold and astringents (Abeanethy). If the tumor is a very vascular one, ligature of the artery leading to it, thus preventing further engorgement of the veins, the primary cause of telangiectasis (and to it these tumors owe their softness when touched by the hand), also are recommended, the introduction of a seton, the hypodermic injection of percloride of iron (Carter has seen death caused by embolism after this; it is, nevertheless, endorsed by different authorities) *; further, the actual cautery, and cautery by medicaments—chloride of zinc. I tried the lapis infernalis in one case—the nævus, a small red speck resembling a broken vein, upon the cheek of a little girl about four years of age, at first increased in size, and then began to diminish, and is now disappearing, about the fourth week after the cauterization. They will often do this, however, if left to themselves. Concentrated nitric acid applied on the point of a pine stick, is probably the most effectual caustic. If we have a medium-sized and soft warty tumor (nævus,), with pedicle, we may pass a silk thread around and strangulate it—cauterizing the wound surface left with argent nit· (lapis). Sir William Ferguson ran two needles through the base of a nævus; these were armed with silk thread and were passed through, the one horizontally, the other perpendicularly; the four ends of the threads left projecting after the needles were withdrawn were then tied together and the nævus thus cut off. If the tumor is a fungus hematodes, the following application will act very well:—

℞. Potas. iodid.,

Aquæ, āā ℥ j. M.

This makes an excellent caustic, and will speedily check the advance of the tumor, if applied every day or two as occasion requires, and contained for some time. If the telangiectasis is of cavernous kind, filled with either blood or lymph, palpitation will give us valuable points, especially as regards fluctuation. If opened, the cavern should be swabbed with a small sponge, wet with a solution of carbolic acid and glycerine, in equal parts, and dressed with

* Bardeleben-Chirurgie, Vol. 1, p. 428.

adhesive plaster. Great care should be exercised if the diagnosis reveals a cavernous tumor. The galvano-caustic is here, above all, the most sure method, and the one that will give the best reselts.

*Diagnosis and Treatment of Glandular Tumors.—Adenoma.*—The diagnosis of adenoma is readily arrived at from hyperplasia of a gland, on which it always sits. Almost all that has been said with reference to healing tumors by operation and pharmaceutical means holds good here, only we may obtain better results with medicaments and compression in adenoma, than in other tumors. One remarkable case that came under personal observation, was that of a man in middle life, whose left breast being affected with adenoma, had swelled to the size of that of a girl of sixteen years; the nipple was regularly elongated, the tumor soft to the touch and altogether like a normal female breast. No treatment was given, as the tumor had become stationery and was not objected to by the patient.

*Polypi*—Medium hard, bright red or yellowish growths, mostly on mucous membranes, from which they hang by a stem. They are covered with cylinder-epithelium externally; toward the centre they present a mass of thickened mucous membrane, having its origin in a hypertrophy of the minute slime or mucous glands. The cause of their formation is usually to be found in chronic catarrhs, and the nose seems to be their first choice. They are of slow growth, and only cause annoyance by a sense of pressure and fullness in the upper part of the nostril, where their stem allows them to slip up and down, and where they may be easily felt and seen, both from the outside and within. Removal is the only cure. This is quickly, easily and safely accomplished by means of a pair of pincers. The patient, seated in a chair should be made to expirate, by which the polypus will descend a little, when it is firmly grasped with the instrument and torn out. The operation is attended with but insignificant loss of blood, and as regards after treatment, nothing is necessary. Very rarely we find one at the top of the pharnyx, which if of a more compact structure, will be best removed by galvano-caustic.

*Sebaceous tumors, cysts, skin tumors, etc.*, are such growths as are contained in dermoid, round sacks, closed, on all sides, the contents being usually either serum or a composition of fat and lime. The sack itself is a part of the tumor. They may also be formed by the encysting of foreign bodies, such as a gunshot or bullet. They are often due to a tendency to fatty degeneration, but little, however, is known about their general etiology. They feel hard and immovable to the touch, are painless and of slow growth. I will select one case out of my own practice as illustrative: G. G., aged 35, male, well developed and healthy, consulted me three years ago about what he supposed to be a shot left in the louble of the ear from the discharge of a gun. Upon examination I told him it must be a shot, as it felt exactly like one in point of size and consistency, and as he had a gun discharged at him, other shot having taken effect at the time. Last month he consulted me again, as I had declined previously to interfere. The tumor was softer, but still right hard to the touch, and had grown to the size of a bird's egg. I operated, making the incision behind the louble of the ear, in order to conceal the scar, and extirpating sack and all. The wound was dressed with adhesive plaster only, and healed rapidly per primam. On opening the sack no shot was found, only sebaceous matter. Operation with the knife is the only radical cure in these cases. A hygroma or gangloin may be punctured, and its contents thus emptied; flunctuation and softness, without inflammation, make then easy to diagnose.—*Med. and Surg. Reporter.*

